# Organizing memories for generalization in complementary learning systems

**DOI:** 10.1038/s41593-023-01382-9

**Published:** 2023-07-20

**Authors:** Weinan Sun, Madhu Advani, Nelson Spruston, Andrew Saxe, James E. Fitzgerald

**Affiliations:** 1 Janelia Research Campus, Howard Hughes Medical Institute, Ashburn, VA USA; 2 Center for Brain Science, Harvard University, Cambridge, MA USA; 3 Department of Experimental Psychology, University of Oxford, Oxford, UK; 4 Gatsby Computational Neuroscience Unit & Sainsbury Wellcome Centre, UCL, London, UK; 5 CIFAR Azrieli Global Scholars Program, CIFAR, Toronto, Ontario Canada

**Keywords:** Network models, Consolidation, Long-term memory, Psychology

## Abstract

Memorization and generalization are complementary cognitive processes that jointly promote adaptive behavior. For example, animals should memorize safe routes to specific water sources and generalize from these memories to discover environmental features that predict new ones. These functions depend on systems consolidation mechanisms that construct neocortical memory traces from hippocampal precursors, but why systems consolidation only applies to a subset of hippocampal memories is unclear. Here we introduce a new neural network formalization of systems consolidation that reveals an overlooked tension—unregulated neocortical memory transfer can cause overfitting and harm generalization in an unpredictable world. We resolve this tension by postulating that memories only consolidate when it aids generalization. This framework accounts for partial hippocampal–cortical memory transfer and provides a normative principle for reconceptualizing numerous observations in the field. Generalization-optimized systems consolidation thus provides new insight into how adaptive behavior benefits from complementary learning systems specialized for memorization and generalization.

## Abstract (web summary)

The authors derive a neural network theory of systems consolidation to assess why some memories consolidate more than others. They propose that brains regulate consolidation to optimize generalization, so only predictable memory components consolidate.

## Main

The brain’s ability to learn, store and transform memories lies at the heart of our ability to make adaptive decisions. Memory is threaded through cognition, from perception through spatial navigation to decision-making and explicit conscious recall. Befitting the central importance of memory, brain regions—including the hippocampus—appear specifically dedicated to this challenge.

The concept of memory has refracted through psychology and neurobiology into diverse subtypes and forms that have been difficult to reconcile. Taxonomies of memory have been drawn on the basis of psychological content, for instance, differences between memories for detailed episodes and semantic facts^1^; on the basis of anatomy, for instance, differences between memories that are strikingly dependent on hippocampus versus those that are not^2^; and on the basis of computational properties, for instance, differences between memories reliant on pattern-separated^3^ or distributed neural representations^4^. Many previous theories have tried to align and unify psychological, neurobiological and computational memory taxonomies^5–8^. However, none has yet resolved long-standing debates on where different kinds of memories are stored in the brain, and, fundamentally, why different kinds of memories exist.

Classical views of systems consolidation, such as the standard theory of systems consolidation^5,9^, have held that memories reside in the hippocampus before transferring completely to the neocortex. Related neural network models, such as the complementary learning systems theory, have further offered a computational rationale for systems consolidation based on the benefits of coupling complementary fast and slow learning systems for integrating new information into existing knowledge^6,10^. However, these theories lack explanations for why some memories remain forever hippocampal-dependent, as shown in a growing number of experiments^2,11^. On the other hand, more recent theories, such as multiple trace theory^7,12^ and trace transformation theory^13^, hold that the amount of consolidation can depend on memory content, but they do not provide quantitative criteria for what content will consolidate, nor why this might be beneficial for behavior.

One possible way forward is to see that memories serve not only as veridical records of experience but also to support generalization in new circumstances^14^. For instance, individual memorized experiences almost never repeat exactly, but they allow us to identify systematic relationships between features of the world, such as ‘ravines predict the presence of water,’ which are common and important for behavior.

Here we introduce a mathematical neural network theory of systems consolidation founded on the principle that memory systems and their interactions collectively optimize generalization. Our theory mathematically defines the generalization performance of an algorithm as its expected error for any possible future input, whether these inputs have been seen in the past or not. This definition is widespread in statistics and machine learning, and it resonates with the intuitive notion that generalizations apply regularities inferred from specific instances to new circumstances. The resulting theory offers new perspectives on diverse experimental phenomena and explains why interaction between multiple brain areas is beneficial. Accurate generalizations require consistent relationships within the environment, and our theory optimizes generalization by using the predictability of memorized experiences to determine when and where memory traces reside. Our results overall propose a quantitative and unified theory of the organization of memories based on their utility for future behavior.

## Results

### Formalizing systems consolidation

**Fig. 1:**
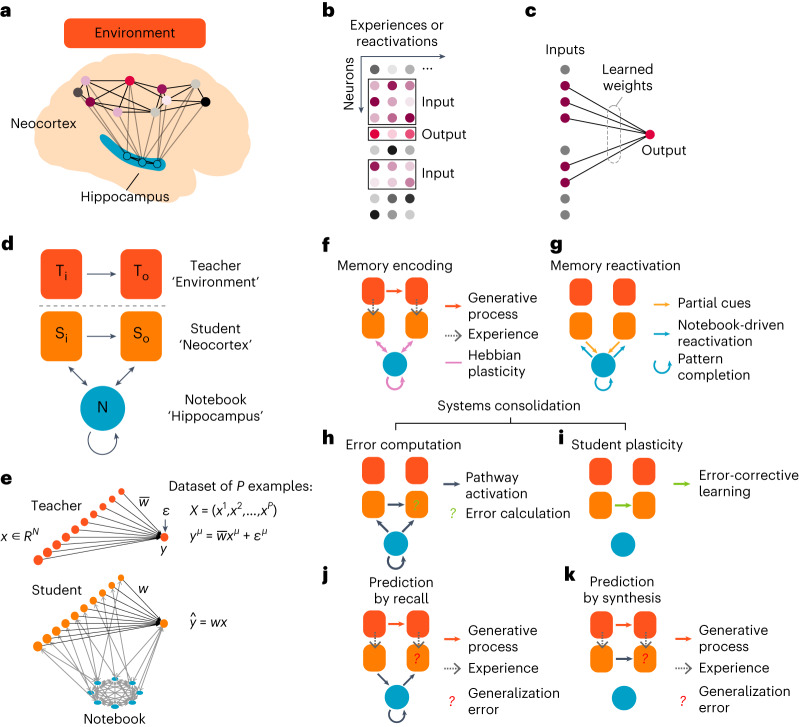
Neural network model of systems consolidation. **a**, Our theoretical framework assumes that the neocortex extracts and encodes environmental relationships within the weights between distributed neocortical neurons in a process mediated by hippocampal reactivation. **b**, Individual experiences or memory reactivations are represented as columns of neuronal activations. We color the output neuron in red, and its activity is determined by the purple input neurons that are connected to it. Neurons that are not connected to the output neuron are colored gray. This example is for illustrative purposes, and an input neuron in one relationship could be an output neuron in another relationship. **c**, Learning modifies weights between the input and output neurons to reproduce each past experience. **d**, Cartoon of the teacher–student–notebook formalism; subscripts ‘i’ and ‘o’ refer to input and output layers. **e**, Neural network model architecture used in most simulations, unless otherwise noted. The teacher is a linear, shallow network with fixed weights that transforms an *N*-dimensional input into a scalar *y*, with a noise term *ε* added to vary the signal-to-noise ratio of the teacher. The student is typically a size-matched network to the teacher, with trainable weights *w*. The notebook is a Hopfield network that is bidirectionally connected to the student that serves as a one-shot learning module for memory encoding and replay (see Methods for details). **f**–**k**, Stages of learning and inferences in the model. The student is activated by each of the teacher-generated examples while the notebook encodes this example through one-shot Hebbian plasticity (**f**). The notebook can reactivate the encoded examples offline and reactivate the student (**g**). The notebook can reactivate previously encoded memories offline to induce memory recall in the student (**h**) and drive student learning (**i**). The student can use either the notebook or internal weights for inference (**j** and **k**). T, teacher; S, student; N, notebook.

We conceptualize an animal’s experiences in the environment as structured neuronal activity patterns that the hippocampus rapidly encodes and the neocortex gradually learns to produce internally^6,10,15,16^ (Fig. 1a). We hypothesize that systems consolidation allows neocortical circuits to learn many structured relationships between different subsets of these active neurons. Focusing on one of these relationships at a time, neocortical circuitry might learn through many experiences (Fig. 1b) to produce the responses of a particular *output* neuron from the responses of other *input* neurons (Fig. 1c). For example, in a human, an output neuron contributing to a representation of the word ‘bird’ might receive strong inputs from neurons associated with wings and flight. In a mouse, an output neuron associated with behavioral freezing might receive strong inputs from neurons associated with the sound of an owl, the smell of a snake or the features of a laboratory cage where it had been shocked.

We first sought to develop a theoretically rigorous mathematical framework to formalize this view of how systems consolidation contributes to learning. Our framework builds on the complementary learning systems hypothesis^6,10^, which posits that fast learning in the hippocampus guides slow learning in the neocortex to provide an integrated learning system that outperforms either subsystem on its own. Here we formalize this notion as a neocortical *student* that learns to predict an environmental *teacher*, aided by past experiences recorded in a hippocampal *notebook* (Fig. 1d). Note that although the theory is centered around hippocampal–neocortical interactions, the core theoretical principles can be potentially applied to other brain circuits that balance fast and slow learning^17–19^.

We modeled each of these theoretical elements with a simple neural network amenable to mathematical analyses (Fig. 1e; Methods). Specifically, we modeled the teacher as a linear feedforward network that generates input–output pairs through fixed weights with additive output noise, the student as a size-matched linear feedforward network with learnable weights^20,21^ and the notebook as a sparse Hopfield network^22,23^. The student learns its weights from a finite set of examples (experiences) that contain both signal and noise. We modeled the standard theory of systems consolidation by optimizing weights for memory. This means that the squared difference between the teacher’s output and the student’s prediction should be as small as possible, averaged across the set of past experiences. Alternatively, we hypothesize that a major goal of the neocortex is to optimize generalization. This means that the squared difference between the teacher’s output and the student’s prediction should be as small as possible, averaged across possible future experiences that could be generated by the teacher.

Learning starts when the teacher activates student neurons (Fig. 1f, gray arrows). The notebook encodes this student activity by associating it with a random pattern of sparse notebook activity using Hebbian plasticity (Methods; Fig. 1f, pink arrows). This effectively models hippocampal activity as a pattern-separated code for indexing memories^24^. The recurrent dynamics of the notebook network implement pattern completion^22,25^, whereby full notebook indices can be reactivated randomly from spontaneous activity or purposefully from partial cues^26^ (Methods; Fig. 1g). Student-to-notebook connections allow the student to provide the partial cues that drive pattern completion (Fig. 1g, orange arrows). Notebook-to-student connections then allow the completed notebook index to reactivate whatever student representations were active during encoding (Fig. 1g, blue arrows). Taken together, these three processes permit the student to use the notebook to recall memories from related experiences in the environment. Thus, our theory concretely models how the neocortex could use the hippocampus for memory recall.

We model systems consolidation as the plasticity of the student’s internal synapses (Fig. 1h,i). The student’s plasticity mechanism is guided by notebook reactivations (Fig. 1h), similar to how hippocampal replay is hypothesized to contribute to systems consolidation^27^. Slow, error-corrective learning aids generalization^28^, and here we adjust internal student weights with gradient descent learning (Fig. 1i). Specifically, we assume that offline notebook reactivations provide targets for student learning (Methods), where the notebook-reactivated student output is compared with the student’s internal prediction to calculate an error signal for learning. We consider models that set the number of notebook reactivations to optimize either memory transfer or generalization. The integrated system can use the notebook (Fig. 1j) or only the learned internal student weights (Fig. 1k) to make output predictions from any input generated by the teacher. We will show that each pathway has distinct advantages for memory and generalization.

### Generalization-optimized complementary learning systems (Go-CLS)

**Fig. 2:**
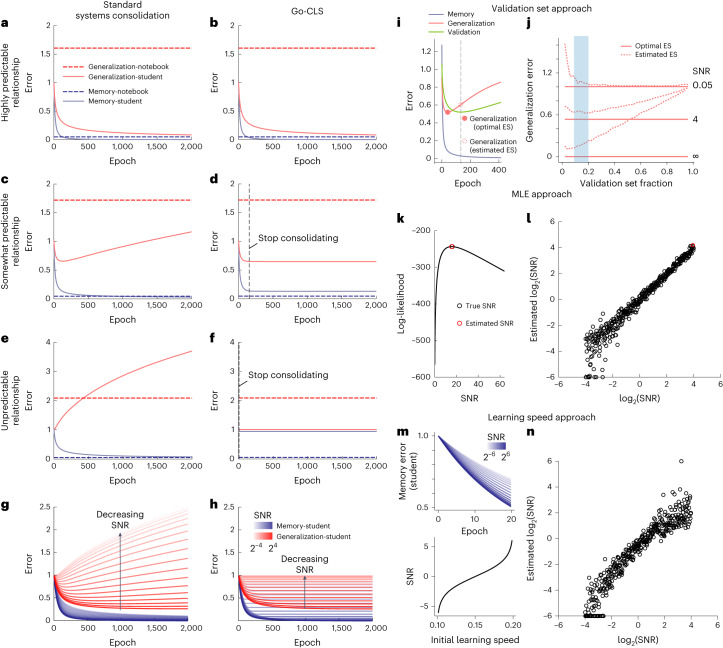
The predictability of experience controls the dynamics of systems consolidation. **a**–**h**, Dynamics of student generalization error, student memorization error, notebook generalization error and notebook memorization error when optimizing for student memorization (**a**, **c**, **e** and **g**) or generalization (**b**, **d**, **f** and **h**) performance. The student’s input dimension is *N* = 100, and the number of patterns stored in the notebook is *P* = 100 (all encoded at epoch = 1; epochs in the *x* axis correspond to time passage during systems consolidation). The notebook contains *M* = 2,000 units, with a sparsity *a* = 0.05. During each epoch, 100 patterns are randomly sampled from the *P* stored patterns for reactivating and training the student. The student’s learning rate is 0.015. Teachers differed in their levels of predictability (**a** and **b**, SNR = ∞; **c** and **d**, SNR = 4; **e** and **f**, SNR = 0.05; **g** and **h**, SNR ranges from 2^−4^ to 2^4^). **i**–**n**, Methods for regulating consolidation. **i**, Using a validation set to estimate optimal early stopping time (SNR = 4, *P* = *N* = 100, 10% of *P* are used as validation set and not used for training). Filled red dot marks the generalization error at the optimal early stopping time (optimal ES), and dashed red dot marks the generalization error at the early stopping time estimated by the validation set (estimated ES). The vertical gray dashed line marks the estimated early stopping time. **j**, Generalization errors at optimal (solid red lines) vs estimated early stopping time (dashed red lines), as a function of the validation set fraction, SNR and *α* (*P*/*N*). The blue shading indicates the validation set fraction from 10% to 20%. **k**, Illustration of maximum likelihood estimation (MLE; Supplementary Information Section 9.2). **l**, MLE predicts SNR well from teacher-generated data. **m**, Initial learning speed monotonically increases as a function of SNR. **n**, Initial learning speed serves as a good feature for estimating true SNR in numerical simulations (*P* = *N* = 1,000).

We next simulated the dynamics of memorization and generalization in the teacher–student–notebook framework to investigate the impact of systems consolidation. We first modeled the standard theory of systems consolidation as limitless notebook reactivations that optimized student memory recall (Fig. 2a,c,e; Methods). Learning begins when the notebook stores a small batch of examples, which are then repetitively reactivated by the notebook in each epoch to drive student learning (Methods). In separate simulations, examples were generated by one of three teachers that differed in their degree of predictability, here controlled by the signal-to-noise ratio (SNR) of the teacher network’s output (Fig. 1e; Methods). The notebook was able to accurately recall the examples provided by each teacher from the beginning (Fig. 2a,c,e, dashed blue lines), and we showed mathematically that recall accuracy scaled with the size of the notebook (Supplementary Information Section 5.2). Notebook-mediated generalization (student in → notebook → student out) was poor for all three teachers (Fig. 2a,c,e, dashed red lines), as rote memorization poorly predicts high-dimensional stimuli that were not previously presented or memorized (Supplementary Information Section 5.3). The student gradually reproduced past examples accurately (Fig. 2a,c,e, solid blue lines), but the signal in each example was contaminated by whatever noise was present during encoding and repetitively replayed throughout learning. Therefore, although the generalization error decreased monotonically for the noiseless teacher (Fig. 2a, solid red line), noisy teachers resulted in the student eventually generalizing poorly (Fig. 2c,e, solid red lines). From a mathematical point of view, this is expected, as the phenomenon of overfitting to noisy data is well appreciated in statistics and machine learning^29,30^.

The implications of these findings for psychology and neuroscience are far-reaching, as the standard theory of systems consolidation assumes that generalization follows naturally from hippocampal memorization and replay; it does not consider when systems consolidation is detrimental to generalization. For example, previous neural network models of complementary learning systems focused on learning scenarios where the mapping from input to output was fully reliable^5,6^. Within our teacher–student–notebook framework, this means that the teacher is noiseless and perfectly predictable by the student architecture. In such scenarios, standard systems consolidation continually improved both memorization and generalization in our model (Fig. 2a, solid red line). However, for less predictable environments, our theory suggests that too much systems consolidation can severely degrade generalization performance by leading the neocortex to overfit to unpredictable elements of the environment (Fig. 2c, solid red line). In highly unpredictable environments, any systems consolidation at all can be detrimental to generalization (Fig. 2e, solid red line). If the goal of systems consolidation is full memory transfer, then our theory illustrates that the system pays a price in the reduced ability to generalize in uncertain environments.

What systems consolidation strategy would optimize generalization? Here we propose a new theory—Go-CLS—which considers the normative hypothesis that the amount of systems consolidation is adaptively regulated to optimize the student’s generalization accuracy based on the predictability of the input–output mapping (Fig. 2b,d,f). For the teacher with a high degree of predictability, the student’s generalization error always decreased with more systems consolidation (Fig. 2b, solid red line), and the student could eventually recall all stored memories (Fig. 2b, solid blue line). Memory transfer, therefore, arises as a property of a student that learns to generalize well from this teacher’s examples. In contrast, a finite amount of consolidation (here modeled by a fixed number of notebook reactivations) was necessary to minimize the generalization error when the teacher had limited predictability (Fig. 2d,f), and our normative hypothesis is that systems consolidation halts at the point where further consolidation harms generalization (Fig. 2d,f, vertical black dashed line). The resulting student could generalize nearly optimally from each of the teachers’ examples (Fig. 2d,f, solid red lines and Supplementary Information Section 7.2), but its memory performance was hurt by incomplete memorization of the training data (Fig. 2d,f, solid blue lines). Nevertheless, the notebook could still recall the memorized examples (Fig. 2b,d,f, dashed blue lines). Go-CLS thus results in an integrated system that can both generalize and memorize by using two systems with complementary properties.

These examples show that the dynamics of systems consolidation models depend on the degree of predictability of the teacher. Therefore, we derived analytical results to comprehensively compare the standard theory of systems consolidation to the Go-CLS theory for all degrees of predictability (Supplementary Information Sections 6 and 7). Standard systems consolidation eventually consolidated all memories for any teacher (Fig. 2g, blue). As anticipated by Fig. 2a–f, the generalization performance varied dramatically with the teacher’s degree of predictability (Fig. 2g, red). Generalization errors were higher for less predictable teachers, and optimal consolidation amounts were lower. Therefore, Go-CLS removed the detrimental effects of overfitting (Fig. 2h, red) but ended before the student could achieve perfect memorization (Fig. 2h, blue, nonzero error). Both the generalization performance and the memory performance improved as the teacher’s degree of predictability increased (Fig. 2h).

Fully implementing this strategy for Go-CLS requires a supervisory process capable of estimating the optimal amount of consolidation (Supplementary Information Section 9). One conceptually simple way to do this is to directly estimate the generalization error dynamics (Fig. 2i,j), which would not require explicit inference of the teacher’s predictability. For instance, the supervisor could divide the notebook’s memorized examples into a training set that drives student learning and a validation set that does not. Because the student’s error on the validation set is an estimate of the generalization error, the supervisor could regulate consolidation by stopping student learning when the validation error starts increasing (Fig. 2i). This strategy works best for relatively small validation sets, as this permits learning from many examples (Fig. 2j).

Another strategy to regulate consolidation is to estimate the predictability of the teacher (Fig. 2k–n). For instance, the supervisor could statistically estimate the teacher’s degree of predictability as the one that maximizes the likelihood of the teacher-generated examples (Fig. 2k). This amounts to comparing the input–output covariance of the teacher-generated data to theoretical expectations, which vary in predictable ways with SNR (Supplementary Information Section 9.2). Alternatively, the supervisor could use the simpler heuristic that the initial learning speed (for a given sized dataset) correlates with predictability (Fig. 2m and Supplementary Information Section 9.3). Each of these methods provides a reasonably accurate estimate of the teacher’s degree of predictability (Fig. 2l,n), which could be used to estimate the optimal early stopping time (Supplementary Fig. 4). Such estimates rely on prior knowledge relating data statistics to the teacher’s degree of predictability, which for more complex environments could be established by meta-learning over developmental, lifelong and evolutionary timescales^31^.

### Relating Go-CLS to diverse experimental results

**Fig. 3:**
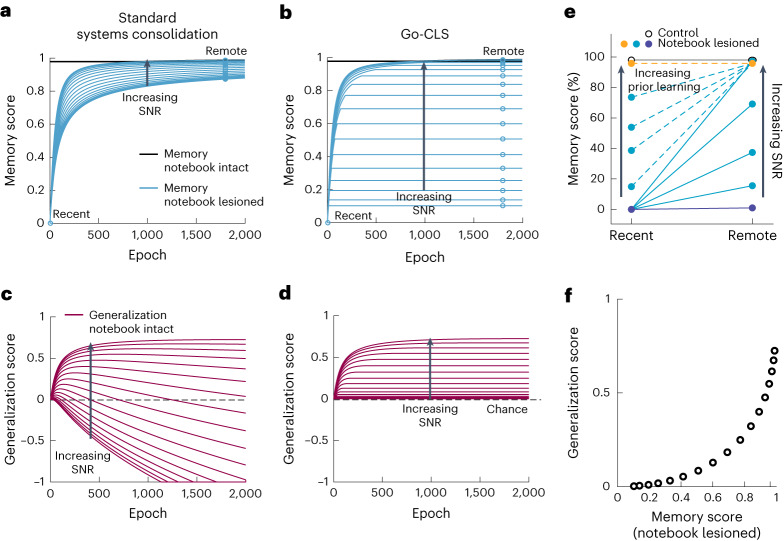
Go-CLS mirrors memory research findings. **a**–**d**, Memorization (**a** and **b**) and generalization (**c** and **d**) scores for the integrated student–notebook system as a function of time and SNR, when optimized for student memorization (**a** and **c**) or generalization (**b** and **d**). Memory and generalization scores are translated from respective error values by score = (*E*_0_ − *E*_*t*_)/*E*_0_, where *E*_0_ and *E*_*t*_ are the generalization or memory errors of a zero-weight student and a trained student at epoch = *t*, respectively. The effect of notebook lesion on memory performance (cyan lines and open circles, open circles simply demarcate the cyan lines at hypothetical ‘recent’ and ‘remote’ timepoints) depended on optimization objective and time (**a** and **b**). **e**, Go-CLS can reproduce a diversity of retrograde amnesia curves (see Methods for model details). **f**, Memory and generalization scores are positively correlated after notebook lesioning.

Experimental literature on the time course of systems consolidation and time-dependent generalization provides important constraints on our theory. We thus sought to model these effects by translating mean square errors (Fig. 2g,h) into memory or generalization scores, where 0 indicates random performance and 1 indicates perfect performance (Fig. 3a–d; Methods). Our framework can use either the student or the notebook to recall memories or generalize (Fig. 1j,k). Here we model the combined system by making predictions with whichever subsystem is more accurate (Methods). This assumption is not critical, as the combined memory (Fig. 3a,b) and generalization scores (Fig. 3c,d) often map onto the notebook and student performances, respectively, but this assumption allows the combined system to switch between subsystems over time (Supplementary Information Section 6.1). Other models might implement more complex memory system selection policies or combine both pathways to obtain statistically better predictions throughout learning. We simulated hippocampal lesions by preventing the combined system from using notebook outputs and ending systems consolidation at the time of the lesion (Fig. 3a,b, cyan). As it takes time for the student to learn accurate generalizations, our systems consolidation models exhibited time-dependent generalization (Fig. 3c,d, purple). In contrast, the notebook permitted accurate memory retrieval from the start (Fig. 3a,b, black).

Standard systems consolidation and Go-CLS theory make strikingly different predictions for how retrograde amnesia depends on the teacher’s degree of predictability (Fig. 3a,b). Researchers usually classify hippocampal amnesia dynamics according to whether memory deficits are similar for recent and remote memories (flat retrograde amnesia), more pronounced for recent memories (graded retrograde amnesia), or absent for both recent and remote memories (no retrograde amnesia; Fig. 3e). As expected^9^, notebook lesions always produced temporally graded retrograde amnesia curves in the standard theory (Fig. 3a). When systems consolidation was instead optimized for generalization, the effects of notebook lesions depended strongly on the predictability of the teacher (Fig. 3b). Therefore, Go-CLS theory can recapitulate a wide diversity of retrograde amnesia curves (Fig. 3e). High- and low-predictability experiences lead to graded and flat retrograde amnesia, respectively (Fig. 3b,e, solid lines). A period of prior consolidation of highly predictable experiences decreases the slope of graded retrograde amnesia (Fig. 3e, dashed light-blue lines), and it’s possible to see no retrograde amnesia at all when the prior consolidation was extensive (Fig. 3e, dashed orange line; Methods). This conceptually resembles schema-consistent learning^32^.

Experiments on time-dependent generalization can also differentiate between the Go-CLS theory and the standard theory. Diverse generalization curves resulted from either model of systems consolidation (Fig. 3c,d), with maximal generalization performance increasing with the predictability of the teacher. However, student overfitting meant that only Go-CLS maintained this performance over time. Standard systems consolidation could even result in a student generalizing maladaptively, resulting in worse-than-chance performance where the trained student interpolates noise in past examples to produce wildly inaccurate outputs (Fig. 3c). Most fundamentally, Go-CLS theory predicts that memory transfer and generalization improvement should be correlated with each other (Fig. 3f), as systems consolidation leads to both. Unpredictable experiences should not consolidate because this would cause maladaptive generalization. Such memories are thus left in their original form and susceptible to strong retrograde amnesia following hippocampal lesion. In contrast, predictable experiences should consolidate and be associated with weak retrograde amnesia and useful learned generalizations.

Go-CLS potentially resolves apparent conflicts in the literature as arising from differing degrees of predictability in the underlying experimental paradigms (Supplementary Information Section 11). This hypothesized correspondence between past experiments and their predictability is intriguing but inconclusive, as it is not yet clear how to quantify the degree of predictability for arbitrary experiments and real-world scenarios. In other words, the theory is consistent with existing findings in principle, but its postdiction of them requires plausible assumptions that may be wrong. Future experiments are critical (see Supplementary Information Section 12 for detailed discussions of experimental tests). Our core theoretical prediction is that the brain optimizes for generalization by regulating the amount of systems consolidation based on the predictability of experience. Direct tests of this prediction require experimental task designs that intentionally vary the degree of predictability and assess the effect on systems consolidation^33^. In addition, experiments that identify the biological mechanisms of predictability estimation and consolidation regulation would be required to establish a comprehensive picture of the neural correspondence of the Go-CLS theory.

### Normative benefits of complementary learning systems for generalization

**Fig. 4:**
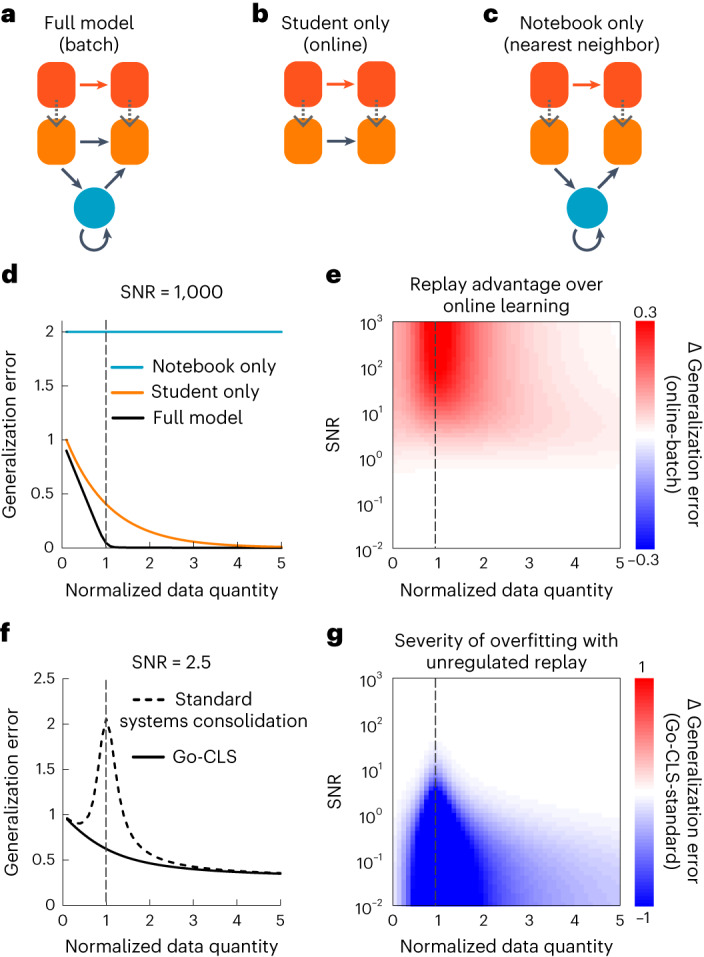
Normative benefits of complementary learning systems for generalization. **a**–**c**, Schematics illustrating learning systems that can use both the student and the notebook (**a**), only the student’s weights (**b**) and only the notebook weights (**c**) for inference. In machine-learning terminology, these systems implement batch learning, online learning and nearest-neighbor regression. **d**, Generalization error as a function of normalized data quantity (or *α*, defined as *α* = *P*/*N*) for each learning system (SNR = 1,000); dashed vertical line indicates *α* = 1. **e**, Advantage of Go-CLS over optimal online learning as a function of SNR and normalized data quantity, measured by the difference in generalization error. **f**, Generalization error as a function of normalized data quantity for the combined system, learning either through Go-CLS or standard systems consolidation (SNR = 2.5). **g**, Severity of overfitting, measured by the difference in generalization error between standard systems consolidation and Go-CLS.

Our framework also provides theoretical insights into the complementary learning systems hypothesis, which posits that hippocampal and neocortical systems exploit fundamental advantages provided by coupled fast and slow learning modules^6,10^. We first investigated its basic premise by comparing generalization in the optimally regulated student–notebook network (Fig. 4a) to what is achievable with isolated student (Fig. 4b) and notebook networks (Fig. 4c). Because the student models the neocortex and the notebook models the hippocampus, these isolated student and notebook networks model learning with only neocortex or only hippocampus, respectively.

Both the degree of predictability and the amount of available data impact the time course of systems consolidation in the student–notebook network (Supplementary Information Sections 6 and 7), so we used our analytical solutions to systematically examine how late-time memory and generalization jointly depend on the amount of training data and degree of predictability (Supplementary Fig. 2). With just a student (Fig. 4b), the system must learn online from each example with no ability to revisit it. This limitation prevented the optimal student-only network from generalizing as efficiently from predictable teacher-generated data as the optimal student–notebook network (Fig. 4d, orange versus black curves), despite modulating its learning rate online to achieve best-case generalization performance (Supplementary Information Section 4.2). We also confirmed that both student-containing networks generalized better than the notebook-only network (Fig. 4d). This is expected because in high dimensions any new random pattern is almost always far from the nearest memorized pattern (Supplementary Information Section 5.3); this is the so-called ‘curse of dimensionality.’

The generalization gain provided by the student–notebook network over the student-only network was most substantial when the teacher provided a moderate amount of predictable data (Fig. 4d,e, dashed vertical line). This result follows because the student–notebook network was unable to learn much when the data were too few or too noisy, and notebook-driven encoding and reactivation of data was unnecessary when the student had direct access to a large amount of teacher-generated data (Supplementary Information Section 4 and 7). Hence, an integrated dual memory system was normatively superior when experience was available, but limited, and the environment was at least somewhat predictable.

The notebook’s ability to replay examples was most advantageous when the number of memorized examples equaled the number of learnable weights in the student (Fig. 4e, dashed vertical line). Remarkably, this amount of data was also the worst-case scenario for overfitting to noise in standard systems consolidation (Fig. 4f,g, dashed vertical line, and Supplementary Fig. 2c). This is similar to the ‘double descent’ phenomenon in machine learning^20,34,35^, where overfitting is worst at an intermediate amount of data related to the network size. Intuitively, neural networks must tune their weights most finely when the number of memorized patterns is close to the maximal achievable number (capacity). This often requires drastic changes in weights to reduce small training errors, producing noise-corrupted weights that generalize poorly. The optimal student–notebook network avoided this issue by regulating the amount of systems consolidation according to the predictability of the teacher. We propose that the brain might similarly regulate the amount of systems consolidation according to the predictability of experiences (Discussion).

### Many facets of unpredictability

**Fig. 5:**
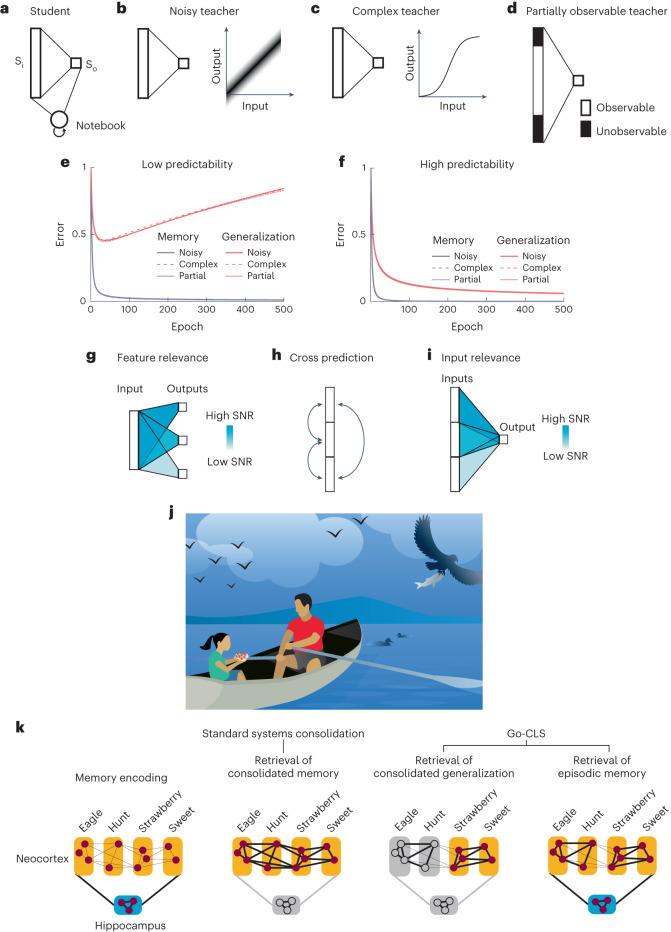
Many forms of unpredictability demand regulated systems consolidation. **a**, The student–notebook learning system. **b**–**d**, Example teachers with unpredictable elements. **b**, A teacher that linearly transforms inputs into noisy outputs. **c**, A teacher that applies a nonlinear activation function at the output unit and cannot be fully predicted by a linear student. **d**, A teacher that only partially reveals the relevant inputs to the learning system. **e**,**f**, Varying predictability within the three different teachers all lead to quantitatively similar learning dynamics (complex teacher implements a sine function at the output unit, see Methods for simulation details). **g**–**i**, The degree of predictability can vary in many ways. For example, the same inputs can differentially predict various outputs (**g**), features can cross-predict each other with varying levels of predictability (**h**) and different learning systems could attend to different teacher features to predict the same output (**i**). **j**, Cartoon illustrating a child’s experience at a lake with her father. **k**, Cartoon illustrating conceptual differences between what is consolidated in standard systems consolidation and Go-CLS.

Our simulations and analytical results show that the degree of predictability controls the consolidation dynamics that optimize generalization. We emphasized the example of a linear student (Fig. 5a) that learns from a noisy linear teacher (Fig. 5b). However, inherent noise is only one of several forms of unpredictability that can cause poor generalization without regulated systems consolidation. For example, when the teacher implements a deterministic transformation that is impossible for the student architecture to implement, the unmodellable parts of the teacher mapping are unpredictable and act like noise (Supplementary Information Section 10). For instance, a linear student cannot perfectly model a nonlinear teacher (Fig. 5c). Similarly, when the teacher’s mapping involves relevant input features that the student cannot observe, the contribution of the unobserved inputs to the output is generally impossible to model (Fig. 5d). This results in unpredictability from the student’s perspective and favors large student networks with enough features to represent the teacher. These sources of unpredictability all consist of a modellable signal and an unmodellable residual (noise; Supplementary Information Section 10), and they yield similar training and generalization dynamics in our model (Fig. 5e,f). The real world is noisy and complicated, and the brain’s perceptual access to relevant information is limited. Realistic experiences thus frequently combine these sources of unpredictability.

All the above-mentioned cases can be generally understood within the framework of approximation theory^36^. The unmodellable part represents a nonzero optimal approximation error for the teacher–student pair. For all these types of generalization-limiting unpredictability, generalization is optimized when systems consolidation is limited for unpredictable experiences. Notably, not all unpredictability limits generalization (Supplementary Information Section 8). For example, independent noise during inference can actually promote generalization, such as in dropout regularization^37^.

Previously we have focused on the scenario of learning a single mapping. All real-life experiences are composed of many components, with relationships that can differ in predictability. Therefore, many relationships must be learned simultaneously, and these representations are widely distributed across the brain. For instance, the same input features may have different utilities in predicting several outputs (Fig. 5g). Furthermore, neocortical circuits may cross-predict between different sets of inputs and outputs (Figs. 1a and 5h), for example, predicting auditory representations from visual representations and vice versa. In this setting, each cross-prediction has its own predictability determined by the noise, the complexity of the mapping, and the features it is based upon. Predictability may also depend on overt and/or covert attention processes in the student. For example, a student may selectively attend to a subset of the inputs it receives (Fig. 5i), making the predictability of the same external experience dependent on internal states that can differ across individuals. This might partially underlie the individual variability in memory consolidation seen in animal behavior^38^. For all the above-mentioned scenarios, Go-CLS theory requires the student to optimize generalization by regulating systems consolidation according to the specific degree of predictability of each modeled relationship contained in an experience. The theory thereby provides a new predictive framework for quantitatively understanding how diverse relationships within memorized experiences should differentially consolidate to produce optimal general-purpose neocortical representations.

## Discussion

The theory presented here—Go-CLS—provides a normative and quantitative framework for assessing the conditions under which systems consolidation is advantageous or deleterious. As such, it differs from previous theories that sought to explain experimental results without explicitly considering when systems consolidation could be counterproductive^5,6,9,11–13^. The central premise of this work is that systems consolidation from the hippocampus to the neocortex is most adaptive if it is regulated to improve generalization, an essential ability enabling animals to make predictions that guide behaviors promoting survival in an uncertain world. Crucially, we show that unregulated systems consolidation results in inaccurate predictions by neural networks when limited data contain a mixture of predictable and unpredictable components. These errors result directly from the well-known overfitting problem that occurs in artificial neural networks when weights are fine-tuned to account for data containing noise and/or unmodellable structure^20,21,29,30,34,35^.

For example, consider the experience of a girl spending a day at the lake with her father (Fig. 5j,k). It may contain predictable relationships about birds flying, swimming and perhaps even catching fish, as well as predictable relationships about fresh-picked strawberries tasting sweet. Our theory posits that these relationships should be extracted from the experience and integrated with memories of related experiences, through regulated systems consolidation, to produce, reinforce and revise predictions (generalizations). On the other hand, unpredictable co-occurrences, such as the color of her father’s shirt matching the color of the strawberries, should not be consolidated in the neocortex. They could nevertheless remain part of an episodic memory of the day, which would permanently depend on the hippocampus for retrieval.

An important but subtle point is that relationships in the environment can be both arbitrary and predictable. For example, consider the case of semantic facts, such as Paris is the capital of France. Although each component of this knowledge is arbitrary, perfect generalization performance is possible. Past experiences indicating that Paris is the capital of France would allow the brain to predict this exact and reliable relationship in future experiences. The learning of reliable semantic facts should be modeled as infinite SNR in our teacher–student–notebook framework.

Go-CLS highlights the normative benefits of complementary learning systems, reveals key concepts that may reconcile previous experimental results (Supplementary Information Section 11) and makes testable predictions that could support or refute the theory (Supplementary Information Section 12). A critical insight from Go-CLS theory is that gradual consolidation of past experiences benefits generalization performance most when experience is limited and relationships are partially predictable (Fig. 4), mirroring ethologically realistic regimes experienced by animals living in an uncertain world. This benefit occurs in a regime where the danger of overfitting is the highest^20,21,34,35^, highlighting the need for a regulated systems consolidation process.

Previous theories have also sought to reconcile these and other experimental observations. For example, multiple trace theory^12^ and trace transformation theory^13^ posit that episodic memories are consolidated as multiple memory traces, with the most detailed components permanently residing in the hippocampus. Contextual binding theory^11^ posits that items and their context remain permanently bound together in the hippocampus. These theories emphasize the role of the hippocampus in the permanent storage of episodic details^2,11–13^, with the neocortex storing less detailed semantic components of memories. In contrast, Go-CLS posits that predictability, rather than detail, determines consolidation. Similarly, Go-CLS favors predictability over frequency, feature overlap or salience as the central determinant of systems consolidation^39–41^.

Our theory has many interesting connections to recent research in artificial intelligence. Go-CLS defines predictability through the optimal approximation error^36^ of a teacher–student pair (Fig. 5a–f). This is distinct from whether optimal student weights can be learned in practice. For example, gradient descent learning dynamics can get stuck in local minima or transiently degrade generalization performance^34,42,43^, but this does not imply that the teacher is unmodellable by the student architecture. Our analytically tractable student cleanly dissociates the optimal approximation error from learning dynamics, but this theoretical distinction becomes impractical when analyzing complex student architectures. Because overfitting is also observed in more complex student architectures (Supplementary Information Section 10.2), as well as in modern deep learning models^34^, we expect that the essential concepts presented here will also apply to broader model classes. However, future research will be needed to determine how the student’s architecture, student’s learning rule and teacher jointly determine the memorization and generalization dynamics achievable by regulated systems consolidation. Similarly, some machine-learning methods can interpolate training data and generalize well^44^, so it would also be interesting to search for student architectures and learning rules that could reduce tension between memorization and generalization. Finally, we’ve focused on simple supervised learning problems; future work should address optimal consolidation in settings that exhibit richer generalization dynamics, such as reinforcement learning^45^ and emergent few-shot learning in large language models^46^.

The fact that an experience’s predictability is a priori unknown has important conceptual implications for regulated systems consolidation. Here we have shown that it’s sometimes possible to accurately infer predictability from data (Fig. 2). This capability allows accurate generalization that is likely critical for building high-fidelity models of the world. However, we do not expect that the brain explicitly implements the schemes as shown in Fig. 2. For instance, it would be surprising if the brain sets aside validation data that never drives learning. Moreover, many studies suggest that the brain relies on suboptimal heuristics for decision-making and other cognitive tasks^47^, and regulating systems consolidation based on inaccurate heuristics could lead to mis-generalization and departures from the predictions of Go-CLS theory. For example, an interesting prediction of Go-CLS theory is that frequent misinformation should be consolidated less than rare gems from a wise source, but this prediction would fail if brains used frequency as a simple heuristic for predictability. Extreme misregulation of consolidation could relate to disorders, such as post-traumatic stress disorder (PTSD)^48^. Modeling regulated systems consolidation in real-world scenarios thus requires a precise understanding of the brain’s predictability estimation algorithm. Targeted experimental tests of Go-CLS theory could avoid this issue by focusing on tasks where animals generalize accurately.

Go-CLS theory does not specify the biological mechanisms by which memory consolidation should be regulated. Given the prominent role of replay in existing mechanistic hypotheses about systems consolidation^27,49^, this would be a natural target for regulation^50–53^. One possibility would be that memory elements reflecting predictable relationships could be replayed together, while unrelated elements are left out or replayed separately. Another would be that entire experiences are replayed, while other processes (for example, attention mechanisms enabled by the prefrontal cortex^[54^) regulate how replayed events are incorporated into neocortical circuits that store generalizations. Neuromodulators are also likely to have important roles. Norepinephrine is hypothesized to represent unexpected changes in the environment^55^, so it could cue the brain to re-estimate the predictability of relationships in the environment. Acetylcholine is proposed to promote memory encoding^56^, suppress replay^57^ and represent stochasticity in the environment^55^. Acetylcholine could, therefore, enable the hippocampus to preferentially encode memories of unpredictable experiences, which would require long-term hippocampal memory traces in Go-CLS theory. Intriguingly, dopamine is known to tag hippocampal memories of rewarding experiences for enhanced replay and consolidation^58,59^. It will be important to determine if acetylcholine or another neuromodulator can similarly tag memories of unpredictable experiences for reduced replay and systems consolidation.

The proposed principle that the degree of predictability regulates systems consolidation reveals complexities about the traditional distinctions between empirically defined episodic and semantic memories^1^. Most episodic memories contain both predictable and unpredictable elements. Unpredictable coincidences in place, time and content are fundamentally caused by the complexity of the world, which animals cannot fully discern or model. Memorizing such unpredictable events in the hippocampus is reminiscent of previous proposals suggesting that the hippocampus is essential for incidental conjunctive learning^60^, associating discontiguous items^61^, storing flexible associations of disparate and distinct elements^62^, relational or configural information^63^ and high-resolution binding^64^. However, our theory holds that predictable components of these episodic memories would consolidate separately to form semantic memories that inform generalization. We anticipate that psychologists and neurobiologists will be motivated by the Go-CLS theory to test and challenge it, with the long-range goal of providing new conceptual insight into the organizational principles and biological implementation of memory.

## Methods

### Teacher–student–notebook framework

Please refer to the Supplementary Information for a detailed description of the teacher–student–notebook framework. The following sections provide a brief description of the framework and simulation details.

### Architecture

The teacher network is usually a linear shallow neural network generating input–output pairs (*x*^*μ*^, *y*^*μ*^), *μ* = 1,···, *P*, through ${y}^{\mu }={{\bar{w}}}{x}^{\mu }+\varepsilon^\mu$, as training examples. Components of the teacher’s weight vector, $\bar{w}$, are drawn i.i.d. from $\mathcal{N} (0,\sigma{^{2}_{w}})$; components of the teacher’s input patterns, *x*^*μ*^, are drawn i.i.d. from $\mathcal{N} (0,1/N)$, where $\mathcal{N}$ is the input dimension and *ε*^*μ*^ is a Gaussian additive noise drawn i.i.d. from $\mathcal{N} (0,\sigma{^{2}_{\varepsilon}})$. The SNR of the teacher’s mapping is SNR = ${\sigma } ^{2}_{w} \slash {\sigma}^{2}_{\varepsilon}$ and we set $\sigma ^{2}_{w} + \sigma^{2}_{\varepsilon} = 1$ to generate output examples of unit variance. For the simulations in Figs. 2–4, the student is a linear shallow neural network whose architecture matches the teacher (both with input dimension = 100 and output dimension = 1). We relaxed this requirement in Fig. 5 to allow mismatch between the teacher and student architectures (Generative models for diverse teachers). Components of the student’s weight vector, *w*, are initialized as zeros (that is, tabula rasa), unless otherwise noted. The notebook is a sparse Hopfield network containing *M* binary units (states can be 0 or 1, *M* = 2,000–5,000 unless otherwise noted). The input and output layers of the student network are bidirectionally connected to the notebook with all-to-all connections.

### Training procedure

All simulations were performed either using MATLAB (2019b) or Python 3. Training starts with the teacher network generating *P* input–output pairs, with certain predictability (SNR), as described above. For each of these *P* examples, the teacher activates the student’s input and output layers via the identity mapping; at the same time, the notebook randomly generates a binary activity pattern, *ξ*^*μ*^, *μ* = 1,···, *P*, with sparsity *a*, such that exactly *aM* units are in the ‘1’ state for each memory. At each of the example presentations, all of the notebook-to-notebook recurrent weights and the student-to-notebook and notebook-to-student interconnection weights undergo Hebbian learning (Supplementary Information Section 1). This Hebbian learning essentially encodes *ξ*^*μ*^ as an attractor state and associates it with the student’s activation (*x*^*μ*^, *y*^*μ*^), for *μ* = 1,···, *P*.

After all *P* examples are encoded through this one-shot Hebbian learning, at each of the following training epochs, 100 notebook-encoded attractors are randomly retrieved by initializing the notebook with random patterns and letting the network settle into an attractor state through its recurrent dynamics. Notebook activations are updated synchronously for nine recurrent activation cycles, and we found that each memory was activated with near uniform probability. Once an attractor is retrieved, it activates the student’s input and output layers through notebook-to-student weights. Because the number of patterns is far smaller than the number of notebook units (*P* < < *M*) in our simulations, the Hopfield network is well below capacity, and most of the retrieved attractors were perfect recalls of the original encoded indices. The reactivation of the student’s output through the notebook, ${\widetilde{y}}^{\mu }$, is then compared to the original output activated by the teacher, *y*^*μ*^, to calculate how well the reactivation resembles the original experience, quantified as the mean squared error. For error-corrective learning, the student uses the notebook reactivated ${\widetilde{x}}^{\mu }$ and ${\widetilde{y}}^{\mu }$. By comparing the student output that is generated from the reactivated input, ${\hat{\widetilde{y}}}^{\mu }{={{w}}\widetilde{x}}^{\mu }$, and the reactivated student output for all *P* examples, the student updates *w* using gradient descent with $\frac{1}{P}{\sum }_{\mu =1}^{P}{({\widetilde{y}}^{\mu }-{\hat{\widetilde{y}}}^{\mu })}^{2}$ as the loss function. The weight update follows:

$$\varDelta w={\mathrm{{learnrate}}}\times \left(\widetilde{Y}{\widetilde{X}}^{T}-w\widetilde{X}{\widetilde{X}}^{T}\right),$$

where $\widetilde{X}$ and $\widetilde{Y}$ are the column-wise stacked matrix form of the 100 reactivated input and output data points, respectively. Training continues for 500–5,000 epochs, and learnrate ranges from 0.005 to 0.1. In our simulations, as long as learnrate is sufficiently small (0.1 or smaller), the results stay qualitatively constant, and the main results do not depend on the specific choices of learnrate. The *P*_test_ number of additional teacher-generated examples, typically 1,000, is used to numerically estimate the generalization error at each time step by $\frac{1}{{P}_{{\mathrm{{test}}}}}{\sum }_{\mu =1}^{{P}_{{\mathrm{{test}}}}}{({{y}_{{\mathrm{{test}}}}}^{\mu }-w{{x}_{{\mathrm{{test}}}}}^{\mu })}^{2}$. For some simulations, we have applied optimal early stopping regularization, where we stop the training when the estimated generalization error reaches a minimum.

### Retrograde amnesia curves

We draw the following connections from network performance in terms of mean squared error to memory and generalization scores, which are typically measured by behavior responses in a task designed to test memorization or generalization performances. When the student weights are zero, the network error corresponds to chance performance in a task, which is typically set as the zero of a memory retrieval metric. As the error decreases with training, the error is related to the memory retrieval score as follows: score = (*E*_0_ − *E*_*t*_)/*E*_0_, where *E* stands for memorization error or generalization error and the subscripts 0 and *t* indicate a zero weight student and a student at time *t* during training, respectively. This is stating that the memory retrieval score at each time point is negatively correlated to the error at that time and normalized into the range of 0 and 1, where 0 indicates chance performance and 1 indicates perfect performance. During memory retrieval (or generalization), the system chooses whichever available module has a lower memorization error (or generalization error). To simulate notebook lesioning at time *t*, the system starts to use only the student for memory recall; in addition, the student’s memory score will remain unchanged with time due to the lack of notebook-mediated systems consolidation. In Fig. 3e, both the SNR and amount of prior learning were varied to produce the diverse shapes of retrograde amnesia curves. For the control simulation, SNR was set to ∞. For the solid retrograde amnesia curves, SNR values were 0.01, 0.1, 0.3, 1 and 8. SNR was set to 50 for the dotted lines simulating the effect of prior consolidation. Each line is a different simulation with the amount of prior consolidation ranging from 8 epochs to 2,000 epochs (learnrate = 0.005). The student size was *N* = 100 and notebook size was *M* = 5,000. For the varying SNR simulations, *P* = 100, and for varying prior consolidation simulations, *P* = 300.

### Generative models for diverse teachers

To explore different ways unpredictability can exist in the environment, we generalize the teacher–student–notebook model by relaxing the linear and size-matched settings to allow for more complex teachers as generative models for producing training data. For the nonlinear teacher setting, a nonlinear activation function is applied to the linear transformation to generate the teacher’s output. A sine function was chosen for the simulation in Fig. 5e. The corresponding noisy teacher’s SNR is numerically determined from the complex teacher’s nonlinearity, as detailed in Supplementary Information Section 10. For the partially observable teacher, the input layer is larger than the student’s, and the student can only perceive a fixed subregion of the teacher input layer. The exact size of the partially observable teacher is set to match the calculated equivalent SNR of the complex teacher.

### Statistics and reproducibility

We provide code to reproduce all simulation results. We did not collect any experimental data for this theoretical study. Therefore, no statistical method was used to predetermine sample size, no data were excluded from the analyses, the experiments were not randomized and the investigators were not blinded to allocation during experiments and outcome assessment.

### Reporting summary

Further information on research design is available in the Nature Portfolio Reporting Summary linked to this article.

## Online content

Any methods, additional references, Nature Portfolio reporting summaries, source data, extended data, supplementary information, acknowledgements, peer review information; details of author contributions and competing interests; and statements of data and code availability are available at 10.1038/s41593-023-01382-9.

## Supplementary information

Supplementary Information Supplementary Figs. 1–7, mathematical analysis and discussion.

Reporting Summary

## Data Availability

The MNIST^65^, CIFAR-10 (ref. ^66^) and Tiny ImageNet^67^ datasets (used in Supplementary Fig. 5) are publicly available from http://yann.lecun.com/exdb/mnist/, https://www.cs.toronto.edu/~kriz/cifar.html and https://www.kaggle.com/c/tiny-imagenet, respectively.

## Code availability

Code reproducing the results is available at GitHub (https://github.com/neuroai/Go-CLS_v2) and archived at Zenodo (10.5281/zenodo.7941122).
